# Biomarkers of the Complement System Activation (C3a, C5a, sC5b-9) in Serum of Patients before and after Liver Transplantation

**DOI:** 10.3390/biomedicines11072070

**Published:** 2023-07-23

**Authors:** Marta Budkowska, Ewa Ostrycharz, Natalia Maria Serwin, Łukasz Nazarewski, Elżbieta Cecerska-Heryć, Marta Poręcka, Paweł Rykowski, Radosław Pietrzak, Krzysztof Zieniewicz, Aldona Siennicka, Beata Hukowska-Szematowicz, Barbara Dołęgowska

**Affiliations:** 1 Department of Medical Analytics, Pomeranian Medical University, Al. Powstańców Wielkopolskich 72, 70-111 Szczecin, Poland; aldona.siennicka@pum.edu.pl; 2 Institute of Biology, University of Szczecin, 71-412 Szczecin, Poland; ewa.ostrycharz@phd.usz.edu.pl (E.O.); beata.hukowska-szematowicz@usz.edu.pl (B.H.-S.); 3 Doctoral School, University of Szczecin, 70-383 Szczecin, Poland; 4 Molecular Biology and Biotechnology Center, University of Szczecin, 71-412 Szczecin, Poland; 5 Department of Laboratory Medicine, Pomeranian Medical University, Al. Powstańców Wielkopolskich 72, 70-111 Szczecin, Poland; natser@pum.edu.pl (N.M.S.); elzbieta.cecerska.heryc@pum.edu.pl (E.C.-H.); barbara.dolegowska@pum.edu.pl (B.D.); 6 Department of General, Transplant and Liver Surgery, Medical University of Warsaw, ul Banacha 1a, 02-097 Warsaw, Poland; lukasz.nazarewski@wp.pl (Ł.N.); mporecka@gmail.com (M.P.); pawel.ryk@gmail.com (P.R.); radekpietrzak93@gmail.com (R.P.); krzysztof.zieniewicz@wum.edu.pl (K.Z.)

**Keywords:** complement system, anaphylatoxins, biomarkers, sC5b-9, liver, transplantation, CRP

## Abstract

The liver has a huge impact on the functioning of our body and the preservation of homeostasis. It is exposed to many serious diseases, which may lead to the chronic failure of this organ, which is becoming a global health problem today. Currently, the final form of treatment in patients with end-stage (acute and chronic) organ failure is transplantation. The proper function of transplanted organs depends on many cellular processes and immune and individual factors. An enormous role in the process of acceptance or rejection of a transplanted organ is attributed to, among others, the activation of the complement system. The aim of this study was the evaluation of the concentration of selected biomarkers’ complement system activation (C3a, C5a, and sC5b-9 (terminal complement complex)) in the serum of patients before and after liver transplantation (24 h, two weeks). The study was conducted on a group of 100 patients undergoing liver transplantation. There were no complications during surgery and no transplant rejection in any of the patients. All patients were discharged home 2–3 weeks after the surgery. The levels of all analyzed components of the complement system were measured using the ELISA method. Additionally, the correlations of the basic laboratory parameters—C-reactive protein (CRP), hemoglobin (Hb), total bilirubin, alkaline phosphatase (ALP), alanine aminotransferase (ALT), aspartate aminotransferase (AST), gamma-glutamyl transpeptidase (GGTP), and albumin—with the parameters of the complement system (C3a, C5a, and sC5b-9) were determined. In our study, changes in the concentrations of all examined complement system components before and after liver transplantation were observed, with the lowest values before liver transplantation and the highest concentration two weeks after. The direct increase in components of the complement system (C3a, C5a, and sC5b-9) 24 h after transplantation likely affects liver damage after ischemia-reperfusion injury (IRI), while their increase two weeks after transplantation may contribute to transplant tolerance. Increasingly, attention is being paid to the role of C3a and CRP as biomarkers of damage and failure of various organs. From the point of view of liver transplantation, the most interesting correlation in our own research was found exactly between CRP and C3a, 24 h after the transplantation. This study shows that changes in complement activation biomarkers and the correlation with CRP in blood could be a prognostic signature of liver allograft survival or rejection.

## 1. Introduction

Approximately 8000 liver transplants are performed each year in the world according to the Organ Procurement and Transplantation Network, and the three-year patient survival outcome is approximately 80%. The increasing number of patients undergoing liver transplantation from year to year implies a global challenge and the need to search for new, more effective, and safer methods of achieving immune tolerance in this group of patients after the transplantation of this organ. It also gives hope to many patients waiting for effective therapy to regenerate the damaged liver earlier and avoid last-resort transplantation. The proper function of the transplanted organ depends on many cellular and immunological processes and individual features. It is believed that factors contributing to the migration, adhesion, differentiation, and survival of cells significantly impact the liver transplant process. Therefore, it is postulated that the presence of an immunologically incompatible organ and its damage in the mechanism of ischemia-reperfusion injury (IRI) may be the result of the impaired activation of chemokines [1,2,3] and growth factors [4,5,6,7,8,9,10,11,12], but primarily the components of the complement system [13,14,15,16,17,18,19,20,21,22,23].

Anaphylatoxins of the complement system contribute to inflammation and adaptive immune responses [13,24]. They can affect the activation of innate immunity cells (dendritic cells, granulocytes, and macrophages) and the secretion of a wide range of cytokines and chemokines. These substances affect the environment of the inflammatory response and direct the response of T lymphocytes, which in turn contributes to the shift of balance towards rejection or acceptance of the transplant [14,15]. The C5a receptor (C5aR) is involved in early allograft inflammatory responses (cellular infiltrates and macrophage accumulation) and allospecific T cell responses in organ recipients [15]. In addition, it is involved in the inflammatory response during ischemic injury, which has a negative impact on the survival of the transplanted organ [25,26]. Anaphylatoxin C3a has the opposite function. Studies have shown that C3a receptor (C3aR) deficiency leads to imbalances between regulatory and effector T cells. On the other hand, C3aR signaling is necessary to generate graft acceptance [16].

Salerno et al. [27] observed changes in the concentration of C3a in a monkey’s heart during the post-transplant period, where recipients had a rapid and significant increase in C3a in plasma after transplantation until the transplant rejection. Pfeifer et al. [17] also studied the plasma concentration of C3a after transplantation over a longer period. The mean level of this anaphylatoxin was significantly increased when compared to those in non-transplanted subjects. Such a significant increase in the C3a concentration suggests classical or lectin pathway activation. Interestingly, after 40–80 weeks, an increase in C3a concentration was noted again (approximately 10,000 ng/mL), with only minor changes in liver function parameters. This could suggest reinfection with the hepatitis C virus or other humoral response [17].

Shah et al. [28] also researched the effect of anaphylatoxins and the function of transplantation. In this study, the median change in plasma C5a levels between 6 and 24 h after transplantation was significantly higher in patients with primary graft dysfunction (PGD) than in patients without PDG. However, there was no significant difference in the change in C3a concentration in persons with and without PGD. An interesting observation is the relationship between the change in C5a levels (between 6 and 24 h after transplantation and before and 6 h after surgery) and the concentration of C3a at 6 h after surgery with an increased risk of death [28].

C5b-9 (membrane attack complex—MAC—C5b-9) is a final product of the complement system and plays an essential role in the immune system [26]. Recent studies indicate that this complex may be involved in the pathogenesis of acute and chronic organ transplant rejection [18]. In the studies of Lautenschlager et al. [19], significant deposition of MAC in neutrophils and lymphocytes during acute organ rejection was observed. They also noted small amounts of MAC in liver cells during rejection. However, they did not report MAC deposition in biopsies taken immediately after transplantation and without clinical rejection symptoms. The complex deposition was not recorded immediately after transplantation, suggesting that MAC activation was not associated with surgery but with a rejection cascade [19]. In the studies of Conti et al. [18], MAC deposition was observed in the extracellular matrix of the “main entries” of the liver lobe and in portal structures in patients with chronic recoil or biliary complications. It is believed that sublitic amounts of MAC in the vascular system, together with anaphylatoxins, play a significant role in the pathomechanism of late and acute transplant rejection [11]. Furthermore, the activation of the complement system and the formation of sC5b-9 is associated with hemodynamic disorders due to abnormal liver function. Moreover, Bellamy et al. [20] showed that after transplant reperfusion, the plasma sC5b-9 concentration increases significantly and returns to the initial values in the later stages of transplantation. Koskinen et al. [21] obtained similar results. They noted an increase in the concentration of C3 and sC5b-9 after reperfusion, with a maximum concentration after 60 min [21].

Many parameters are necessary for the clinic to monitor the liver and the patient’s condition after transplantation. In the face of the above facts and public health, the aim of this study was to assess the changes in the concentration of selected components of the complement system, molecules directly related to the activity of the nonspecific immune response, in the serum of 100 patients before and after liver transplantation (24 h, two weeks). At this stage of the research, we decided to determine the levels of three biomarkers of complement system activation (C3a, C5a, and sC5b-9), which are involved in initiating inflammatory processes that may lead to rejection of the transplanted organ. Additionally, the correlations of the basic laboratory parameters with the parameters of the complement system (C3a, C5a, and sC5b-9) were determined. 

## 2. Materials and Methods

### 2.1. Study Group 

The study was conducted on a group of 105 patients (45 women and 60 men) aged 20 to 68 years undergoing liver transplantation at the Department and Clinic of General, Transplant, and Liver Surgery of the Medical University of Warsaw. There were no complications during surgery and no transplant rejection in 100 patients (44 women, 56 men). During the planned collection period, 4 out of 5 patients did not live to the second time point, i.e., 24 h after transplantation, while the last patient died and did not live to the third time point (2 weeks after transplantation). All patients without complications were discharged home 2–3 weeks after the surgery. Demographic and laboratory data on analyzed patients are summarized in Table 1. This study was approved by The Bioethical Commission at the Warsaw Medical University (No. KB/224/2016). 

### 2.2. Study Material

To determine the examined factors, approximately 8 mL of venous blood was collected from patients in tubes with a clotting activator to obtain a serum fraction. The material was collected immediately before liver transplant surgery, on the first day after surgery, and two weeks after transplantation. The peripheral blood was then centrifuged and transferred to new tubes and kept at −80 °C until the samples were transported on dry ice to the Medical Analytics Department of the Pomeranian Medical University, where all analyses were performed.

### 2.3. Assay Procedure

The concentration of selected components of the complement system was measured in all samples, both before and after liver transplantation. For C3a and C5a, it was necessary to evaluate the levels of their breakdown products, C3a-desArg and C5a-desArg, because both C3a and C5a are unstable in vivo and are very quickly converted by endogenous carboxypeptidase N to these less active but much more stable forms [29]. Therefore, levels of C3a-desArg, C5a-desArg, and sC5b-9 were determined using the enzyme-linked immunosorbent assay (ELISA) reagent kits (Human C3a ELISA Kit, BD OptEIA ™, Human C5a ELISA Kit II, BD OptEIA™, and Human C5b-9 ELISA Set, BD OptEIA™, respectively). All procedures were conducted according to the manufacturer’s instructions. Products of the reaction were measured using the EnVision microplate reader (Perkin Elmer, Waltham, MA, USA) at 450 nm, and their concentration was calculated based on standard curves. 

CRP was determined by an immunoturbidimetric assay in the serum on the Cobas 6000 module c501 biochemistry analyzer from Roche (Roche, Rotkreuz, Switzerland). Other parameters, such as total bilirubin, ALP, ALT, AST, GGTP, and albumin, were determined in the serum using the spectrophotometric method and the Cobas 6000 module c501 biochemistry analyzer from Roche (Roche, Switzerland). Similarly, optical detection in EDTA whole blood determined Hb using the MEK-7300 hematology analyzer (Nihon Kohden, Tokyo, Japan). All determinations were performed in the Central Laboratory of the University Clinical Center of the Medical University of Warsaw according to the manufacturer’s instructions at the same time points (before transplantation (0 h), 24 h after transplantation, and 2 weeks after transplantation).

### 2.4. Statistical Analysis

The results were evaluated using the RStudio program and the Statistica PL 13 statistical package (StatSoft, Kraków, Poland). The Anderson–Darling test was performed to assess the distribution of variables. Due to the fact that the compared factors were time-related variables, the analysis of their differences was based on paired tests. The Friedman ANOVA test and the Kendall compliance test were used to determine possible changes in all tested factors’ concentrations. The Wilcoxon signed-rank test was performed to assess the differences in parameter concentrations. Additionally, the correlations of basic laboratory parameters (CRP, Hb, total bilirubin, ALP, ALT, AST, GGTP, and albumin) with the tested components of the complement system were performed using Spearman’s correlation test.

## 3. Results

### 3.1. Analysis of Average C3a-desArg Concentrations in Patients before (0 h) and after Liver Transplantation (24 h, Two Weeks)

Our studies showed that the highest C3a-desArg concentrations occur two weeks after liver transplantation (5869 ng/mL), while the lowest occur directly before transplantation (4639 ng/mL) (Table 2).

**Figure 1:**
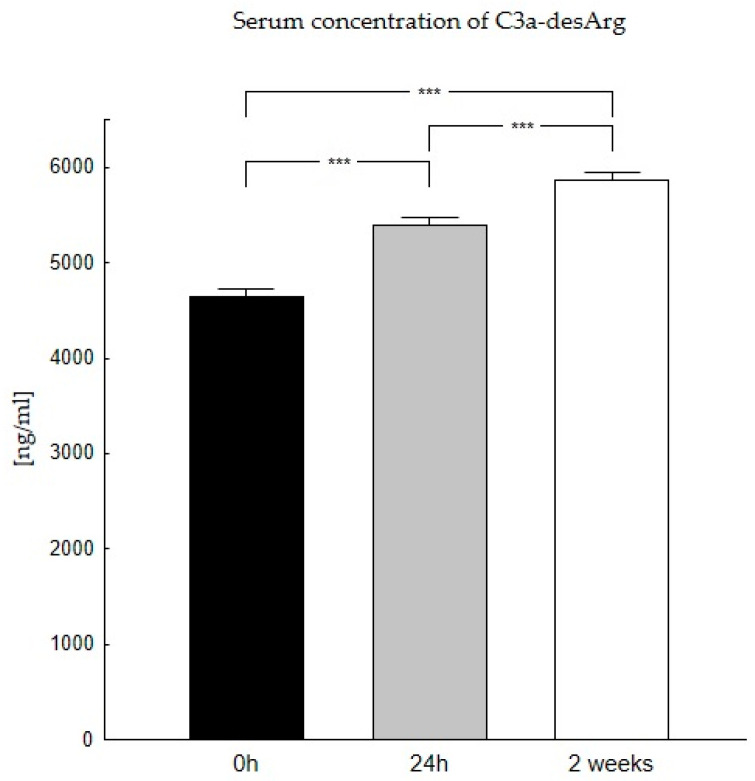
Concentration of C3a-desArg in serum in patients in the period before (0 h) and after the transplantation (24 h, 2 weeks). Serum concentration of C3a-desArg was the lowest at 0 h and increases with time after transplantation. C3a-desArg concentration was evaluated using an ELISA assay. Data were compared with the Friedman ANOVA test and the Kendall test. The Wilcoxon signed-rank test was performed to assess the differences in parameter concentrations. *p*-values below 0.05 were considered statistically significant. Bars indicate the mean ± standard error of the mean (SEM), *** *p* < 0.0001.

Our research confirmed the occurrence of significant differences in C3a-desArg concentrations in the period before and after the transplantation. The Wilcoxon signed test confirmed the presence of statistically significant differences in the concentrations of this anaphylatoxin before and after liver transplantation (*p* < 0.0001) (Figure 1).

### 3.2. Analysis of Average C5a-desArg Concentrations in Patients before (0 h) and after Liver Transplantation (24 h, Two Weeks)

**Figure 2:**
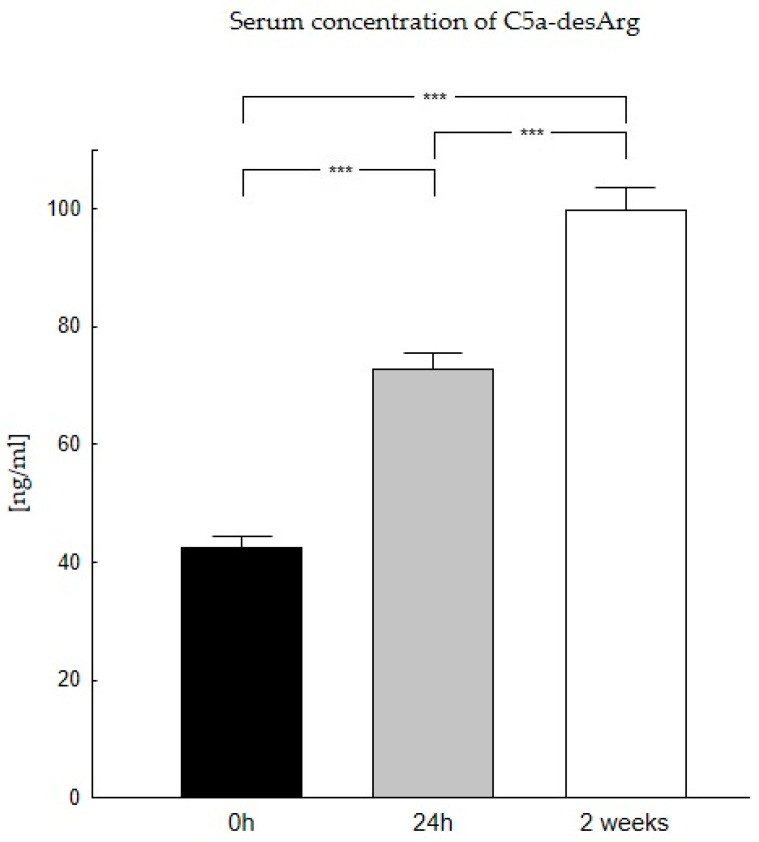
Concentration of C5a-desArg in serum in patients in the period before (0 h) and after the transplantation (24 h, 2 weeks). Serum concentration of C5a-desArg was the lowest at 0 h and increases with time after transplantation. C5a-desArg concentration was evaluated using an ELISA assay. Data were compared with the Friedman ANOVA test and the Kendall test. The Wilcoxon signed-rank test was performed to assess the differences in parameter concentrations. *p*-values below 0.05 were considered statistically significant. Bars indicate the mean ± standard error of the mean (SEM), *** *p* < 0.0001.

Our studies showed that the highest C5a-desArg concentrations occur two weeks after liver transplantation (100 ng/mL), while the lowest occur before transplantation (42 ng/mL) (Table 2). The analysis confirmed differences in C5a-desArg concentrations in the period before and after the transplantation. The Wilcoxon signed-rank test confirmed the presence of statistically significant differences in the concentrations of this anaphylatoxin before and after liver transplantation (*p* < 0.0001) (Figure 2).

### 3.3. Analysis of Average sC5b-9 Concentrations in Patients before (0 h) and after Liver Transplantation (24 h, Two Weeks)

**Figure 3:**
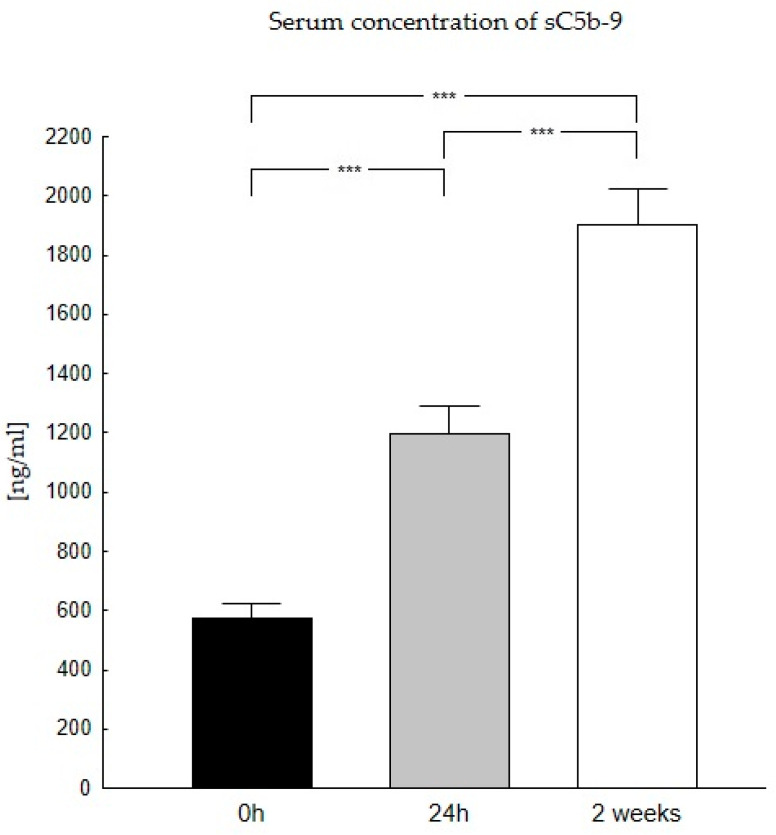
Concentration of sC5b-9 in serum in patients in the period before (0 h) and after the transplantation (24 h, 2 weeks). Serum concentration of sC5b-9 was the lowest at 0 h and increases with time after transplantation. sC5b-9 concentration was evaluated using an ELISA assay. Data were compared with the Friedman ANOVA test and the Kendall test. The Wilcoxon signed-rank test was performed to assess the differences in parameter concentrations. *p*-values below 0.05 were considered statistically significant. Bars indicate the mean ± standard error of the mean (SEM), *** *p* < 0.0001.

Our studies showed that the highest sC5b-9 concentrations occur two weeks after liver transplantation (1901 ng/mL), while the lowest occur before transplantation (574 ng/mL) (Table 2). The analysis confirmed differences in the sC5b-9 concentration in the period before and after transplantation. The Wilcoxon signed test confirmed statistically significant differences in the concentrations of this complement product before and after liver transplantation (*p* < 0.0001) (Figure 3).

### 3.4. Analysis of All Average Concentrations in Deceased Patients

During the planned collection period, four out of five patients did not live to the second time point, i.e., 24 h after transplantation, while the last patient died and did not live to the third time point (2 weeks after transplantation). Demographic and laboratory data on analyzed patients are summarized in Table 3.

Our studies on the five patients showed that the concentrations of C3a, C5a, and sC5b-9 before transplantation were 6408 ng/mL, 66 ng/mL, and 985 ng/mL, respectively (Table 4). Only one of them survived to the second time point but did not live to the third time point (2 weeks after transplantation). The results of this patient 24 h after transplantation were as follows: 6378 ng/mL for C3a-desArg, 64 ng/mL for C5a-desArg, and 1327 ng/mL for sC5b-9. Due to the very small group of deceased patients, it was impossible to perform statistical analyses.

### 3.5. Correlations of Basic Laboratory Parameters with Complement System Parameters (C3a, C5a, and sC5b-9)

As part of the statistical analysis, Spearman’s correlation test was performed for laboratory parameters (CRP, Hb, total bilirubin, ALP, ALT, AST, GGTP, and albumin) with the tested complement system components (C3a, C5a, and sC5b-9).

No correlation was observed with any of the components of the complement system tested in the case of ALP, total bilirubin, GGTP, and albumin.

**Figure 4:**
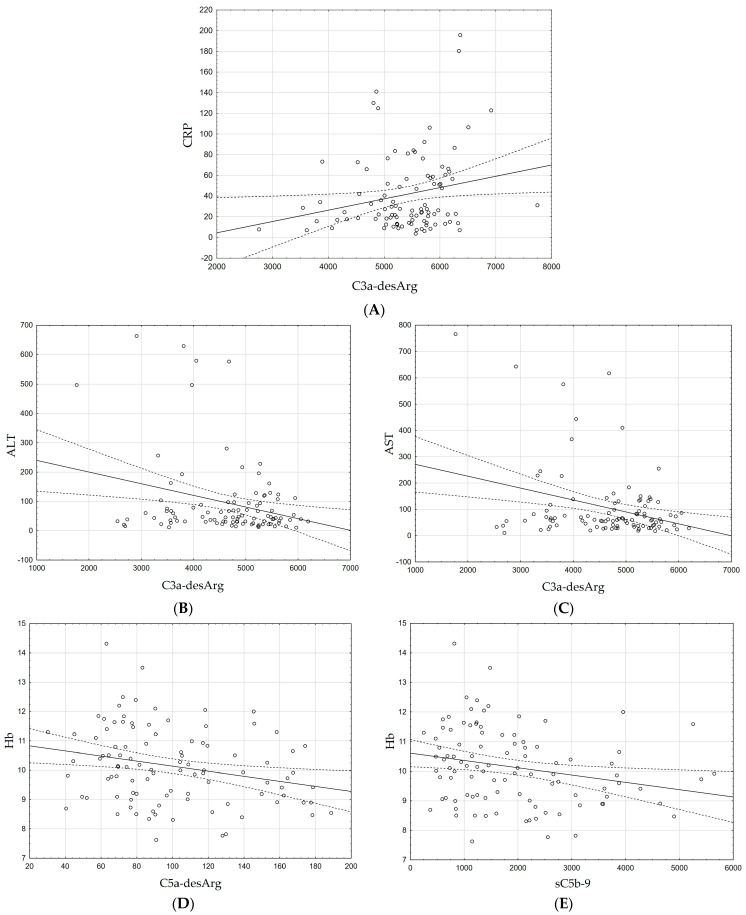
Correlation between basic laboratory parameters with complement system parameters was performed by Spearman’s correlation. (**A**) The positive correlation apart between concentration of CRP and the concentration of C3a 24 h after liver transplantation (r = 0.2188, *p* = 0.029). (**B**) The negative correlation apart between ALT and the concentration of C3a before transplantation (r = −0.2735, *p* = 0.006). (**C**) The negative correlation between AST and the concentration of C3a before transplantation (r = −0.3070, *p* = 0.002). (**D**) The negative correlation for Hb 2 weeks after transplantation with the levels of C5a (r = −0.2557, *p* = 0.010). (**E**) The negative correlation for Hb 2 weeks after transplantation with the levels of sC5b-9 (r = −0.2375, *p* = 0.017). Concentration of complement components (C3a-desArg, C5a-desArg, and sC5b-9) was evaluated using an ELISA assay. CRP was determined by immunoturbidimetric assay in serum on the biochemistry analyzer, while liver enzymes (ALT and AST) and Hb were determined by spectrophotometry method and biochemistry analyzer in serum and in EDTA whole blood on the hematology analyzer, respectively. *p*-values below 0.05 were considered statistically significant.

In the case of CRP, we did not find any statistically significant correlations, apart from one positive correlation, between the concentration of CRP and the concentration of C3a 24 h after liver transplantation (r = 0.2188, *p* = 0.029) (Figure 4A).

In the cases of ALT and AST, we observed negative correlations between the levels of these liver enzymes and the concentration of C3a before transplantation (r = −0.2735, *p* = 0.006 for ALT, r = −0.3070, *p* = 0.002 for AST) (Figure 4B,C). 

A negative correlation was also observed for hemoglobin 2 weeks after transplantation with the levels of C5a (r = −0.2557, *p* = 0.010) and sC5b-9 (r = −0.2375, *p* = 0.017) in the same period (Figure 4D,E).

## 4. Discussion

Contemporary achievements in the field of transplantology have become possible primarily due to dynamically developing experiments and scientific research. Organ transplantation is currently a life-saving operation for people with end-stage multiple organ failure. Some specialists predict that in a few years, transplantation will be every tenth operation carried out in the world. Currently, the liver is one of the most commonly transplanted organs, and the number of these operations is growing from year to year. Due to so many liver transplantations, it is crucial to determine the mechanisms responsible for the proper function of this organ after transplantation. Over the years, researchers have shown that one of the most important mechanisms affecting the process of organ transplantation, including the liver, is the selected components of the complement system [13,14,15,16,17,18,19,20,21,22,23,26]. 

Our studies, assessing the changes in complement components before and after liver transplantation, showed that all examined factors’ highest concentrations were observed two weeks after transplantation. In turn, the lowest values were recorded right before transplantation. The lowest concentration of components of the complement system before transplantation likely does not result from a defect of complement activation but a decrease in the synthesis of its factors as a consequence of progressive liver damage but from many diseases of this organ, including viral hepatitis B or C (HBV, HCV) or autoimmune diseases such as primary biliary cirrhosis (PBC) [17,24,30]. 

Ronholm et al. [22] studied changes in C3a, C5a, and C5b-9 levels before transplantation, one minute before reperfusion, and 1–2 min, 5 min, 30 min, and 6–12 h after the beginning of reperfusion. As in our studies, the lowest concentration of the studied factors was observed before transplantation, but they did not report any significant changes in C3a levels before the reperfusion of the transplanted liver. C3a and C5b-9 levels were elevated in every case after restoring blood flow compared to preoperative concentrations, but the highest level was recorded after 30 min. Levels of C5a during surgery remained unchanged when compared to the pre-operative concentration. Our determinations were performed using ELISA and covered a much larger study group, while Ronholm et al. [22] included 16 people and the concentration of C3a and C5a antigens were measured by radioimmunoassay (RIA). Additionally, we measured the C5b-9 concentration, while Ronholm et al. indicated its biological activity. Therefore, our observations may result from a different measurement method of the studied factors and different sizes of the study group. Furthermore, the differences in the highest concentrations may arise from different times of blood collection. Ronholm et al. studied complement components only 6–12 h after reperfusion. In contrast, in our study, the last blood collection was two weeks after transplantation, and during this period, an immune reaction activating the complement system could have occurred. According to our observations and the results obtained by Ronholm et al. [22], the reason that the concentration of complement components increases after liver transplantation likely lies in the act of transplantation itself [31].

The research shows that all three pathways of the complement system may play an essential role in the IRI mechanism in the newly transplanted liver. Activation of C3 and C5, and consequently the anaphylatoxins C3a and C5a, triggers the inflammatory response and formation of sC5b-9 [32]. Increasing evidence indicates that sC5b-9 exerts several pro-inflammatory responses acting directly on endothelial cells to stimulate transendothelial migration of leukocytes and may contribute to promoting and/or enhancing inflammation [33,34]. Additionally, anaphilatoxin may affect the secretion of soluble chemotactic factors that recruit inflammatory cells [35]. Studies show that the activation of the complement system during liver transplantation is important from pathophysiological and clinical points of view. sC5b-9 levels reflect complement activation and its negative impact on hepatocytes and suggest a correlation between the level of sC5b-9 activation and the intensity of changes in the post-operative levels of AST and factor V. Local complement activation may also play an indirect role in the pathogenesis of graft damage [36,37].

The balance between immunity and tolerance usually determines the acceptance or rejection of a transplanted organ [13]. Studies on animal models have provided convincing evidence that C3a and C5a anaphylatoxins play an essential role in T lymphocyte-mediated cellular immunity. C5a has been shown to have a positive effect on transplant tolerance, in most cases [25,38]. At the same time, C3a is involved in the development of Th2 cell responses [25]. The role of C3a and C5a in the development of Th17 cells is also controversial because these anaphylatoxins demonstrate both inhibitory and stimulating effects on these lymphocytes [25,38]. Therefore, an increase in C3a and C5a levels after transplantation, together with reaching their highest concentrations two weeks after, may be related to the formation of complement-dependent graft tolerance. However, due to the inconsistent literature data, this issue should be investigated more thoroughly. To assess the concentration of complement components and the transplant rejection biomarkers in these patients, long-term (half a year or a year) post-transplantation studies should be performed. At the same time, in vitro studies on the T-cell progenitor cell line should also be carried out to assess the effect of C3a and C5a anaphylatoxins on the direction of T lymphocyte development.

We would also like to pay attention to studies on a small group of patients who died in the peri-transplant period that show a tendency in which the concentrations, regardless of the tested component of the complement system, are initially higher than in the group of patients without complications. It is quite interesting, but we did not decide to perform statistical analyses due to the very small research group, and in the future, we will plan to perform these analyses on a larger group of deceased patients. On the other hand, based on our previous studies in a group of healthy people [39], we also noticed that the results of patients without complications in the post-transplantation period in the case of C3a and sC5b-9 are most similar 2 weeks after transplantation, but still lower than in healthy people. We suppose that these concentrations may level out later when the immune system and the liver itself are fully regenerated after the transplant. In this case, it would be reasonable to monitor these patients in the later post-transplantation period to capture such changes in concentrations. However, such an observation would be significantly impeded due to the fact that these patients came from all over Poland, and at the time of accepting the transplant, they were already discharged and monitored in transplant clinics in their cities or midwives’ cities closest to their place of residence.

In our research, we also noticed a few interesting correlations of laboratory indicators with the components of the complement system we studied. In the cases of ALT and AST, we observed negative correlations between the levels of these liver enzymes and the concentration of C3a before transplantation. The liver enzymes AST and ALT are important biomarkers of liver damage or cirrhosis. Due to their intracellular nature during disease, they are released into the serum due to necrosis and apoptosis of hepatocytes [40]. The C3 protein is primarily synthesized by the liver. Therefore, during liver damage, reduced levels of C3 protein hydrolysis products may be observed, such as C3a. In addition, in some diseases, antigen–antibody complexes may form, leading to the activation of the complement system and causing excessive consumption of its components [41,42]. A negative correlation was also observed for hemoglobin 2 weeks after transplantation with the levels of C5a and sC5b-9 in the same period. The incidence of anemia, according to the WHO definition, in patients undergoing liver transplantation was high at 73% [43]. Increased levels of sC5b-9 can suggest enhanced complement activation, which can lead to erythrocyte hemolysis resulting in anemia [44].

From the point of view of liver transplantation, the CRP correlation with C3a, occurring 24 h after the liver transplantation, seems to be the most interesting. CRP is an acute-phase protein, synthesized by the liver, the synthesis of which begins approximately 6 h after the inflammatory injury [45].CRP is also considered to be an inexpensive, easy-to-measure, but potentially non-specific biomarker of immune system activation [46,47]. Its serum levels can be used as a general indicator of increased production of cytokines, especially interleukin-6, the production of which is induced, among others, by IL-1β, IL3, or tumor necrosis factor [48]. The surgical procedure, which is the transplantation of this organ, on the one hand, induces the increase in CRP in response to a strong pro-inflammatory stimulus, and on the other hand, its production may also depend on the functional capacity of the allogeneic transplant [49]. A damaged graft or delayed normalization of its function can prevent it from responding to inflammation properly, delaying or even interrupting CRP production. Studies have shown that a blunted increase in CRP in the first postoperative day may be an indicator of poor allograft function and is associated with hospital mortality [50,51]. Among our patients, amid whom we did not observe any complications during or after surgery, we noticed a statistically significant (*p* <0.0001) more than 3-fold increase in CRP levels 24 h after transplantation, compared to the baseline level (Table 1), which proves that in their case, there was no blunting of the increase in CRP.

In addition, increasing attention is being paid to the role of C3a and CRP as biomarkers of damage and failure of various organs. Gombos et al. [52] show that elevated C3a levels are associated with biomarkers of acute phase reactions and inflammation, including CRP during ischemic heart disease, and may predict the severity of heart failure and death of patients [52]. C3a may also serve as a good biomarker in acute pancreatitis. C3a correlated well with CRP; however, it showed greater specificity, and when tested together with sC5b-9, also better sensitivity. Therefore, the researchers point out that measuring C3a levels is a sensitive parameter and may be a better alternative to CRP for predicting severe acute pancreatitis [53]. Similar observations were made by Wolbink et al. [54] in patients with sepsis. Plasma C3a levels correlate significantly with CRP levels. In addition, studies have shown that part of complement activation is due to an endogenous mechanism involving CRP and correlates with the morbidity and mortality of patients. Therefore, simultaneous testing of C3a and CRP may be a good biomarker of tissue damage [54]. CRP also exhibits its own pro-inflammatory effect, activating the complement system in a classical way and stimulating NK cells [55,56]. Interestingly, an I/R study in rats showed that activated C3, sC5b-9, and CRP colocalize in hepatocytes, suggesting that CRP is a main mediator of classical complement activation during I/R [57]. On the other hand, Li et al. [58] showed that CRP inhibits C3a-dependent and neutro-phil-dependent amplification of inflammation in the injured liver and may limit destructive complement activation in acute liver injury [58]. From a scientific point of view, but also from the point of view of post-transplantation diagnostics, the study of the correlation between C3a and CRP seems to be a good biomarker of the occurrence of an overreaction of the immune system to the transplanted organ or damage to the transplanted liver. The positive correlation of CRP with C3a 24 h after transplantation, when the inflammatory response is strongest, may suggest the role of C3a in enhancing the mobilization of inflammatory cells, which is necessary to generate the acceptance of the transplanted organ.

## 5. Conclusions

The lowest concentrations of the studied factors—C3a-desArg, C5a-desArg, and sC5b-9—occur right before transplantation and then grow to reach the highest values two weeks after. The lowest pre-transplantation values are a consequence of the primary disease causing the reduction in the synthesis of components of the complement system. We suspect that transplantation itself activates the complement system, which causes a direct increase in the concentration of its components and may lead to liver damage caused by the IRI mechanism. In turn, we assume the highest concentrations of the factors found two weeks after transplantation may contribute to the emergence of transplant tolerance, but this aspect requires further research. Therefore, we wonder whether the observation of changes in the concentration of complement system components could help monitor the function of the transplanted liver after surgery. In addition, the correlation of CRP with C3a 24 h after transplantation may suggest that the latter protein may behave as an acute phase protein in a transplant and may be an additional biomarker of the stratification risk of transplant acceptance/rejection, and in our particular case, the prognostic signature of liver allograft survival. There is no doubt that a better understanding of the role of the various components of the complement system and its interaction with other parameters could help develop new therapies that suppress complement components and improve graft survival.

## Figures and Tables

**Table 1:** Demographic and laboratory features of analyzed patients.

Time of Blood Collection			
Parameters	0 h	24 h	2 Weeks
Gender (M/F)	56/44	56/44	56/44
Age (mean ± SEM)	49 ± 1.4	49 ± 1.4	49 ± 1.4
CRP (mean ± SEM, NR < 5 mg/L)	12.2 ± 1.46	41.5 ± 3.76	17.3 ± 1.93
Hb (mean ± SEM, NR 12–16 g/dL)	11.8 ± 0.22	10.3 ± 0.13	10.1 ± 0.13
Total bilirubin (mean ± SEM, NR 0.2–1.1 mg/dL)	5.9 ± 0.91	3.1 ± 0.32	1.8 ± 0.31
ALP (mean ± SEM, NR 40–120 IU/L)	161 ± 11.8	267 ± 15.3	235 ± 20.3
ALT (mean ± SEM, NR 5–40 IU/L)	95 ± 13.4	300 ± 22.6	128 ± 13.0
AST (mean ± SEM, NR 5–40 IU/L)	107 ± 13.6	111 ± 15.1	48 ± 5.7
GGTP (mean ± SEM, NR 5–40 IU/L)	148 ± 25.8	759 ± 50.5	539 ± 47.6
Albumin (mean ± SEM, NR 3.8–4.2 g/dL)	3.6 ± 0.07	3.1 ± 0.04	3.4 ± 0.05

Abbreviations: 0 h—before transplantation; 24 h—24 h after transplantation; 2 Weeks—2 weeks after transplantation; M—men; F—women; CRP—C-reactive protein; Hb—hemoglobin; ALP—alkaline phosphatase; ALT—alanine aminotransferase; AST—aspartate aminotransferase; GGTP—gamma-glutamyl transpeptidase; SEM—standard error of the mean; NR—normal range.

**Table 2:** Descriptive data for levels of C3a-desArg, C5a-desArg, and sC5b-9 before (0 h) and after transplantation (24 h, 2 weeks).

Parameters	C3a-desArg [ng/mL]			C5a-desArg [ng/mL]			sC5b-9 [ng/mL]		
Time of Blood Collection	0 h	24 h	2 Weeks	0 h	24 h	2 Weeks	0 h	24 h	2 Weeks
Minimum	1763	2757	3668	11	24	30	105	213	250
Maximum	6194	7746	9693	96	171	189	3463	5600	5647
Median	4818	5503	5872	40	71	90	438	900	1466
Mean	4639	5399	5869	42	73	100	574	1196	1901
Standard error of the mean	92	75	75	1.9	2.9	3.8	50	95	124
Lower quartile	3981	5036	5439	27	52	70	269	542	967
Upper quartile	5351	5850	6217	54	85	121	668	1572	2534
Interquartile range	1369	814	779	27	33	51	399	1030	1567

**Table 3:** Demographic and laboratory features of analyzed deceased patients (*n* = 5).

Parameters	0 h	24 h	2 Weeks
Gender (M/F)	4/1	0/1	0/0
Age (mean ± SEM)	50 ± 1.5	53	-
CRP (mean ± SEM, NR < 5 mg/L)	15.1 ± 0.83	70.2	-
Hb (mean ± SEM, NR 12–16 g/dL)	11.5 ± 0.12	10.1	-
Total bilirubin (mean ± SEM, NR 0.2–1.1 mg/dL)	6.7 ± 0.33	4.8	-
ALP (mean ± SEM, NR 40–120 IU/L)	153 ± 6.7	298	-
ALT (mean ± SEM, NR 5–40 IU/L)	148 ± 5.7	350	-
AST (mean ± SEM, NR 5–40 IU/L)	161 ± 4.4	169	-
GGTP (mean ± SEM, NR 5–40 IU/L)	202 ± 11.2	849	-
Albumin (mean ± SEM, NR 3.8–4.2 g/dL)	3.5 ± 0.04	2.9	-

Abbreviations: 0 h—before transplantation; 24 h—24 h after transplantation; 2 Weeks—2 weeks after transplantation; M—men; F—women; CRP—C-reactive protein; Hb—hemoglobin; ALP—alkaline phosphatase; ALT—alanine aminotransferase; AST—aspartate aminotransferase; GGTP—gamma-glutamyl transpeptidase; SEM—standard error of the mean; NR—normal range.

**Table 4:** Descriptive data for levels of C3a-desArg, C5a-desArg, and sC5b-9 in deceased patients before transplantation (0 h, *n* = 5).

Parameters	C3a-desArg [ng/mL]	C5a-desArg [ng/mL]	sC5b-9 [ng/mL]
Time of Blood Collection	0 h	0 h	0 h
Minimum	5256	47	655
Maximum	7154	96	1868
Median	6710	59	777
Mean	6408	66	985
Standard error of the mean	65	1.9	45
Lower quartile	6175	49	695
Upper quartile	6743	79	927
Interquartile range	568	30	232

## Data Availability

The data presented in this study are available on request from the corresponding author. The data are not publicly available due to institutional privacy restrictions.
